# Enzymatic Properties and Mutational Studies of Chalcone Synthase from *Physcomitrella patens*

**DOI:** 10.3390/ijms13089673

**Published:** 2012-08-03

**Authors:** Raja Noor Zaliha Raja Abdul Rahman, Iffah Izzati Zakaria, Abu Bakar Salleh, Mahiran Basri

**Affiliations:** 1 Faculty of Biotechnology and Biomolecular Sciences, Enzyme and Microbial Technology Research Group, Universiti Putra Malaysia, Selangor 43400, Malaysia; E-Mails: milounited_86@yahoo.com (I.I.Z.); abubakar@biotech.upm.edu.my (A.B.S.); 2 Faculty of Science, Universiti Putra Malaysia, Serdang 43400, Malaysia; E-Mail: mahiran@science.upm.edu.my

**Keywords:** chalcone synthase, site-directed mutagenesis, active site, by-products

## Abstract

*Pp*CHS is a member of the type III polyketide synthase family and catalyses the synthesis of the flavonoid precursor naringenin chalcone from *p*-coumaroyl-CoA. Recent research reports the production of pyrone derivatives using either hexanoyl-CoA or butyryl-CoA as starter molecule. The Cys-His-Asn catalytic triad found in other plant chalcone synthase predicted polypeptides is conserved in *Pp*CHS. Site directed mutagenesis involving these amino acids residing in the active-site cavity revealed that the cavity volume of the active-site plays a significant role in the selection of starter molecules as well as product formation. Substitutions of Cys 170 with Arg and Ser amino acids decreased the ability of the *Pp*CHS to utilize hexanoyl-CoA as a starter molecule, which directly effected the production of pyrone derivatives (products). These substitutions are believed to have a restricted number of elongations of the growing polypeptide chain due to the smaller cavity volume of the mutant’s active site.

## 1. Introduction

Flavonoids and isoflavonoids are important plant secondary metabolites that mediate diverse biological functions such as chemical messengers and antioxidants. One of the main key enzymes involved in the flavonoid biosynthesis is chalcone synthase (CHS). Chalcone synthase catalyzes the condensation of p-coumaroyl-CoA with three C_2_ units from malonyl-CoA producing a naringenin chalcone [1]. The synthesis of naringenin chalcone, from which diverse flavonoid end products are derived, led to extensive studies that have been done involving hundreds of CHS genes cloned and characterized from different types of plants [2,3].

However, there is a lack of studies on the enzymatic properties and regulation of chalcone synthase (CHS) in bryophytes. Mosses (Musci, Bryophytae) are one of the oldest groups of land plants. Their life cycle mainly consists of a simple photoautotrophic haploid gametophytic generation. The potential of mosses as a model system in studying higher plant biological processes has led to the first genomic sequence of *Physcomitrella patens* [4]. Moreover, *P. patens* is the first moss to be successfully transformed and has recently been singled-out as the first land plant. Therefore, in this study, the CHS gene (Acession No: DQ 275627.2) was cloned from the gametophore tissues of *P. patens* due to the availability of the *P. patens* EST and genomic sequences.

Moreover, recent studies on the molecular and physiological responses of *P. patens* to ultraviolet-B (UV-B) radiation showed an accumulation of quercetin derivatives (phenolic compound), possibly representing a component of the UV sunscreen. Further analysis showed an accumulation of the CHS and Phenylalanine ammonia lyase (PAL) gene families upon induction of the UV-B radiation [5]. Therefore, in-depth understanding of the potential key genes, particularly the CHS gene, in regulating the response to UV-B is crucial to unveil the evolutionary aspects of UV-B tolerance of *P. patens* towards stress factors.

The chalcone synthase superfamily synthesizes various secondary metabolites in bacteria, fungi and plants. These metabolites played an important role during the early evolution of land plants by providing protection against various environmental stresses such as the earlier mentioned UV irradiation. The genome of the moss *P. patens* contains 17 putative CHS superfamily genes [6]. Chalcone synthase (CHS E.C. 2.3.1.74) catalyzes the first committed step of flavonoid biosynthesis. Chalcone synthase catalyzes the condensation reaction of *p*-coumaroyl-CoA and three acetate units of malonyl-CoA producing naringenin chalcone [1].

Chalcone synthase is a homodimer of 40–4000 kDa subunits containing a Cys-His-Asn catalytic triad in the active site. The polyketide formation reaction is initiated with the loading of a starter molecule (CoA esters) into the active site Cys, which is followed by malonyl-CoA decarboxylation, polyketide chain elongation, and cyclization of the enzyme-bound polyketide intermediate [7]. Theoretically, several factors contribute to the molecular diversity of the polyketide products such as the selection of the starter molecule, the number of polyketide chain elongations, and the mechanism of the cyclization and aromatization reaction. Interestingly, only a small modification of the active site is required to generate a remarkable functional diversity of the type III polyketide synthase (PKSs) [8].

It has been found that the *P. patens* chalcone synthase (*Pp*CHS) is capable of utilizing various aliphatic CoA esters as starter molecules to form a variety of tetraketide-intermediates [9]. Recent work has reported the formation of pyrone derivatives as one of the *Pp*CHS reaction products using hexanoyl-CoA and butryl-CoA as starter molecules [10]. It has been suggested that the reaction product was likely to be due to premature termination of two condensation reactions [11]. Similar results were obtained by chalcone synthase from *Petroselinum hortense* when tested with both butryl-CoA and hexanoyl-CoA, which produced chalcone analogues such as phlorobutyrophenone and phlorocaprophenone [12].

Moreover, three-dimensional studies of chalcone synthase revealed that the size of the cavity in the active site determines the starter molecule selectivity and the upper limit of the chain length in the polyketide products [13]. Thus, we expected that site-directed mutants of *Pp*CHS might provide an insight into the relation between the active site cavity and the conversion of products produced from non-physiological starter molecules (hexanoyl-CoA).

## 2. Results and Discussions

### 2.1. Purification and Characterization of Recombinant Chalcone Synthase

**Figure 1:**
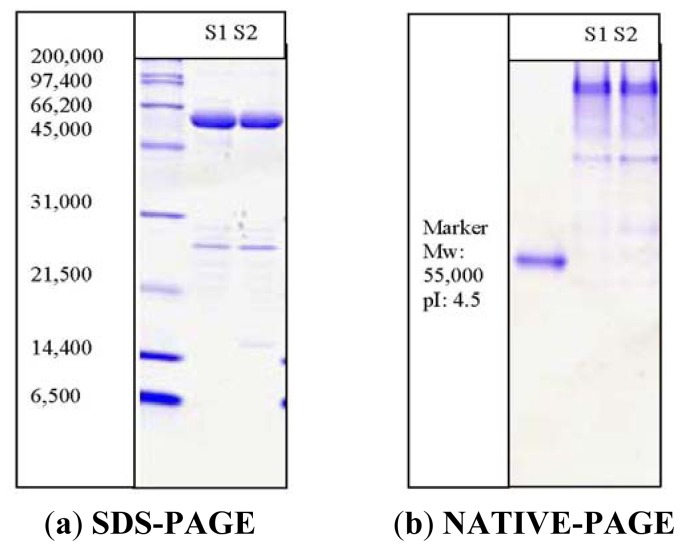
SDS-PAGE (12%) of *Pp*CHS after affinity chromatography. M: standard protein markers were β-galactosidase (116 kDa), bovine serum albumin (66.2 kDa), ovalbumin (45 kDa), lactate dehydrogenase (35 kDa), restriction endonuclease Bsp 981(25 kDa), and β-lactoglobulin (18.4 kDa). Lane 1(S1) and lane 2(S): purified fractions of *Pp*CHS. The *Pp*CHS was expressed as a thioredoxin (Trx)-fusion protein (~62 kDa) along with a Histidine-tagged and *S*-tagged.

A 1.2 kb open reading frame of the *Pp*CHS (Acession No: DQ 275627.2) was cloned in the BL21 (*DE3*) plysS using a PET32 (a) vector system. Specific primers [Forward primer (5′-CGGG CCA TTG AA ATG GCT TCT GCT-3′) and Reverse primer (5′-GAA TTC T CTA AGC GGA GTT GGGG-3′)] were designed to amplify the full-length of the open reading frame (ORF). The PpCHS was expressend as a thioredoxin (Trx)-fusion protein (~62 kDa) along with a Histidine-tag, which allows a single purification step if affinity chromatography using Ni^2+^ Sepharose resin is to be carried out. Thioredoxin is a 12-kD oxidoreductase enzyme containing a dithiol-disulfide active site, which may increase with the soluble, active, properly folded target protein by facilitating the formation of disulphide bonds in the cytoplasm. The purity of the purified *Pp*CHS was analyzed through SDS-PAGE and NATIVE-PAGE as shown in Figure 1. The isoelectric point (pI) of *Pp*CHS was determined to be approximately 7.1, however, the purified protein showed better migration under acidic conditions (pI 4.5) of NATIVE-PAGE. Apart from this, a molecular weight of approximately 120 kDa for PpCHS was determined by gel filtration (Sephacryl 200). The molecular weight of 62 kDa obtained on the SDS-PAGE indicates that the PpCHS is composed of two subunits.

Chalcone synthase catalyzes the formation of naringenin chalcone from *p*-coumaroyl-CoA and malonyl-CoA [1]. It catalyzes the condensation reaction of three acetyl units from malonyl- CoA with p-coumaroyl-CoA to produce naringenin chalcone [1]. However, recent reports have shown that *Pp*CHS is capable of accepting hexanoyl-CoA as a starting molecule to produce hydroxyl-pyrone intermediates [10]. Reactions with different substrates such as hexanoyl-CoA were found to yield pyrone by-products, perhaps due to premature two condensation reactions between hexanoyl-CoA and malonyl-CoA [11]. Another type of polyketide synthase (PKS) from *Cannabis sativa* was also tested using hexanoyl-CoA as a substrate. It has been suggested that this enzyme converts one molecule of hexanoyl-CoA and three molecules of malonyl-CoA into olivetol. The proposed mechanism relates the formation of olivetol to Aldol-type cyclization [14]. Therefore, in this current work, hexanoyl-CoA was used as an alternative substrate to characterize and investigate the enzymatic properties of *Pp*CHS.

Several factors, such as the time and the substrate concentration, were investigated. Based on the production of the cyclization products, only 37% relative activity was obtained using hexanoyl-CoA as a substrate relative to the *p*-coumaroyl-CoA (100%) catalyzed reaction [1]. Furthermore, due to the low conversion rates when using hexanoyl-CoA, the concentration of the substrates needed to maximize the production of the products was investigated.

**Figure 2:**
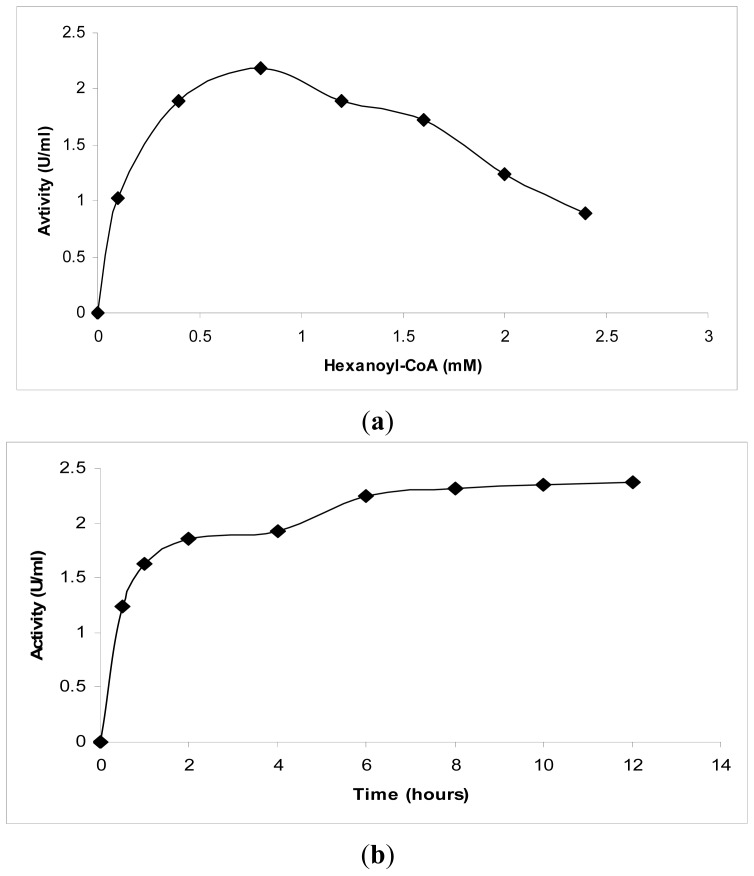
Kinetic studies on chalcone synthase activity using hexanoyl-CoA. (**a**) The effect of the substrate concentration on chalcone synthase activity; (**b**) The effect of the incubation time on chalcone synthase activity.

Several different concentrations of hexanoyl-CoA (0.1 mM to 2.4 mM) were tested, as shown in Figure 2(a). These experiments revealed that 0.8 mM hexanoyl-CoA exhibited the highest production of products. No further increment was observed at higher substrate concentrations. In addition, the effect of the incubation time on the amount of product formed was studied. The results obtained revealed a significant increase after 30 min of incubation (Figure 2(b)). Likewise, the reaction of PKS from *Cannabis sativa* with hexanoyl-CoA showed no significant increase in the amount of product after 45 min of incubation [14].

Generally, chalcone synthase is known for its pH dependent activity. Therefore, the impact of these changes on the pyrone formation was determined. Interestingly, with hexanoyl-CoA as the substrate, the highest activity (in terms of pyrone formation) was found at pH 8, which is different from the optimum pH using p-coumaroyl-CoA, which is pH 7. Previous work has reported the formation of various chalcone analogs from butyryl-CoA and hexanoyl-CoA at pH 6.5, but no products were detected at pH 8.0 [12].

**Figure 3:**
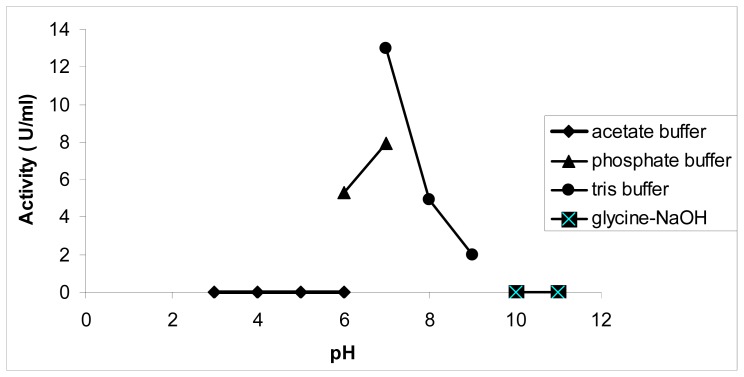
Effect of pH on chalcone synthase activity. *Pp*CHS was assayed at various pHs from pH 4 to pH 12. Acetate buffer was used for pH 3 to 6, sodium phosphate buffer was used for pH 6 to 7, Tris-HCl buffer was used for pH 7 to pH 9, and glycine-NaOH buffer was used for pH 10 to 11. 0.4 mM hexanoyl-CoA was used as a substrate, and the production of pyrone analogs was quantified as activity (U/mL).

As is shown in Figure 3, at lower pHs, between 3 and 5, and at higher pHs, between 9 and 11, pyrone analogs are not produced, but other by-products were detected. Another polyketide synthase from *Cannabis sativa* produced olivetol from hexanoyl-CoA in a potassium phosphate buffer with pH 6.8 [14].

**Figure 4:**
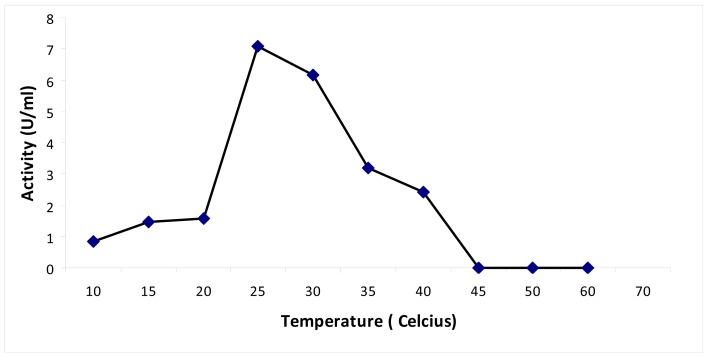
Thermal stability of chalcone synthase. Thermal stability of *Pp*CHS was carried out in the range of 10 to 60 °C.

The recombinant *Pp*CHS exhibited an optimum temperature of 30 °C (results not shown). A similar result was obtained for PKS from *Cannabis sativa*, for which the incubation reaction was perfomed at 30 °C [14]. A thermal stability study was performed by pre-incubating the chalcone synthase for 30 min at different temperatures in the range of 10 °C to 60 °C prior to the enzyme assay. According to the results obtained (Figure 4), the chalcone synthase activity increased at every 5 °C interval as the temperature increased up to 25 °C. At temperatures higher than 35 °C, there was a decrease in enzyme activity.

### 2.2. Mutational Studies to Study Factors That Effect the Substrate Specificity of PpCHS

The three-dimensional structure of chalcone synthase from *Medicago sativa* shows the four main catalytic residues of the active site (Cys 164, His 303, Asn 336, Phe 215), conserved in other CHS-like enzymes [7]. In the crystal structure of CHS from *Medicago sativa*, the Cys 164 residue forms a hydrogen bond with the His 303 residue with an estimated distance of 3.5 Å. In the case of *Pp*CHS, the predicted three-dimensional structure showed a calculated distance of 4 Å between the Cys 170 and His 309. Earlier, it had been proposed that the Cys 164 acted as a nucleophile, while His 303 acted as a general base by abstracting protons from Cys 164 to form a reactive thiolate for chalcone formation. Later on, through several mutational studies, the possibility of a stable imidazolium-thiolate ion pair formation between these two residues was suggested.

Apart from this, most mutational and chemical modification studies carried out to investigate the catalytic Cys-His interaction were often analyzed based on the production of naringenin chalcone using *p*-coumaroyl-CoA as a starting ester [15]. However, chalcone synthase’s unique characteristic is its ability to use various starter CoA esters. As previously reported, chalcone synthase from *Physcomitrella patens* exhibits 37% preference toward hexanoyl-CoA [1]. Therefore, this work describes the effect of Cys 170 Arg and Cys 170 Ser substitutions on the binding of other non-physiological starter CoA esters into the *Pp*CHS’s catalytic cavity based on the production of pyrone derivatives.

**Figure 5:**
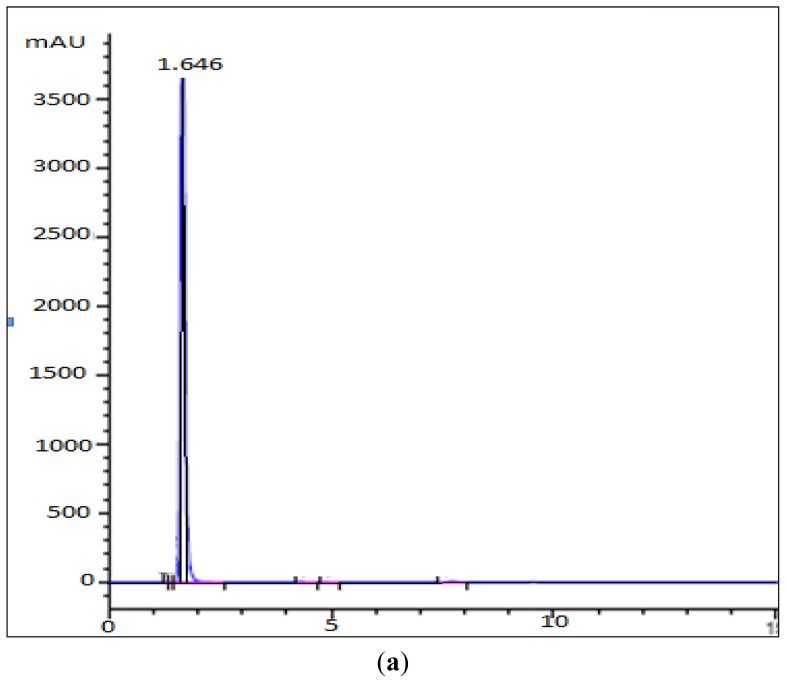

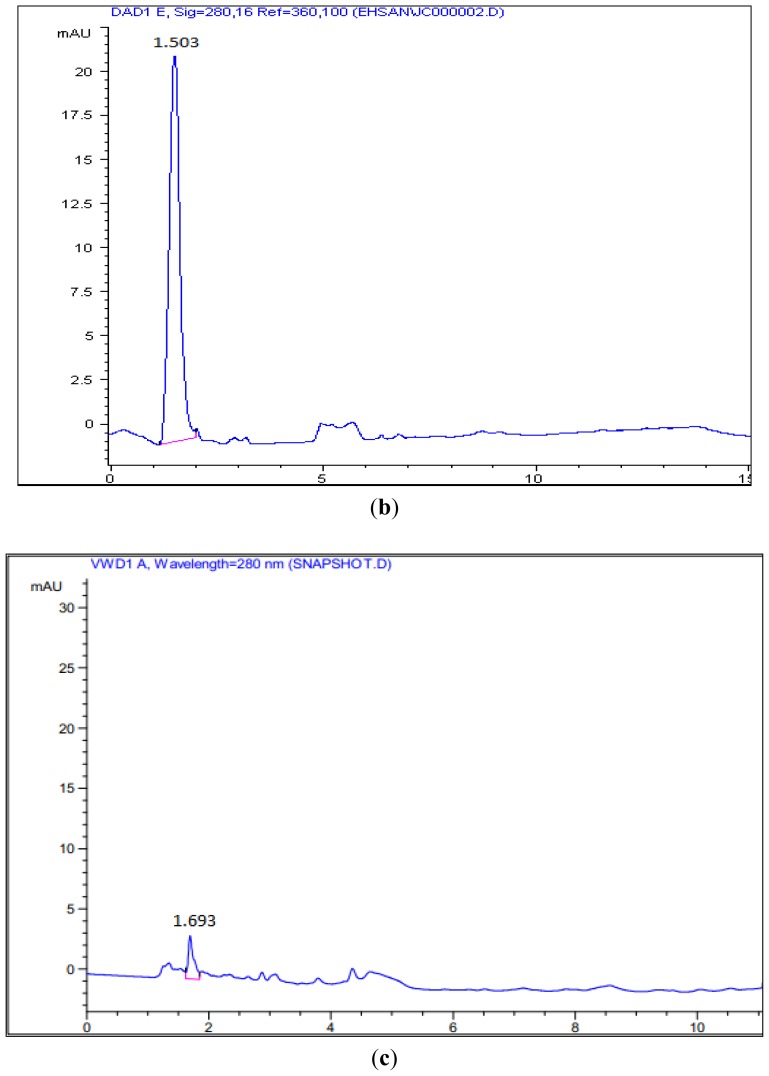

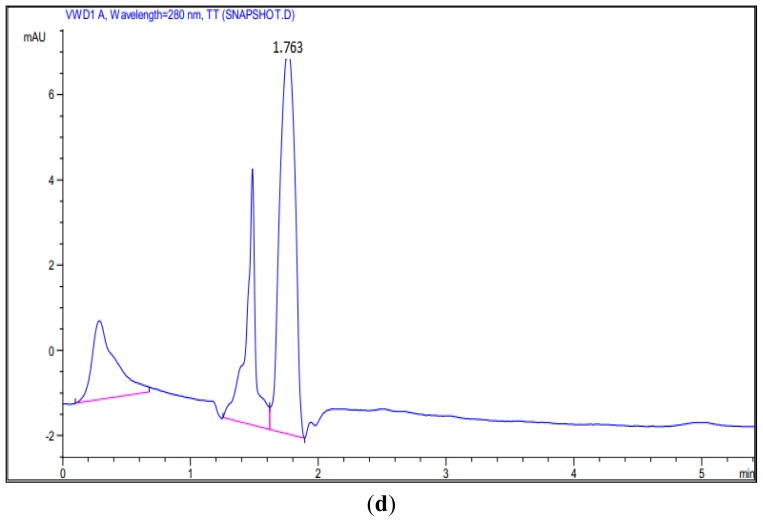
HPLC Analysis of the reaction of hexanoyl-CoA and malonyl-CoA with PpCHS, Cys 170 Ser mutant, and Cys 170 Arg mutant. (**a**) 4-hyrdoxy-6-methyl-2-pyrone (Standard), Retention time: 1.65; (**b**) Reaction product of PpCHS, Retention time: 1.503; (**c**) Reaction product of Cys 170 Ser mutant, Retention time: 1.693; (**d**) Reaction product of Cys 170 Arg mutant, Retention time: 1.763.

**Figure 6:**
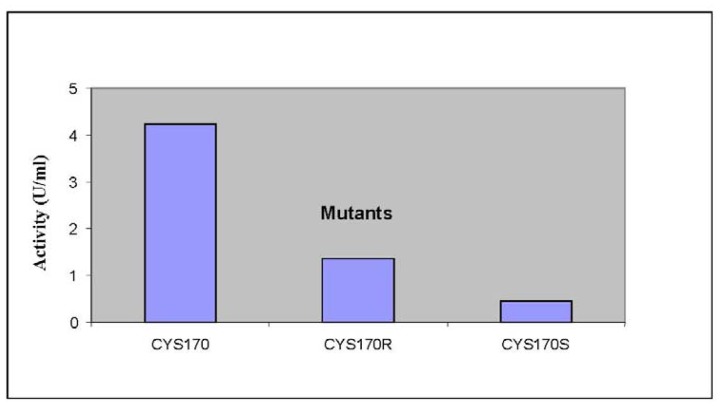
The production of reaction products by *Pp*CHS mutants. The product formation of *Pp*CHS mutants was performed with 0.4 mM hexanoyl-CoA using the standard chalcone synthase assay. The activity (U) is defined as the amount (pmol) of product produced per second.

Previously, it was found that *Pp*CHS utilizes hexanoyl-CoA, producing significant amounts of polyketide products. In this study, HPLC analyses were carried out to quantify the new product formed. Based on the retention time of a 4-hydroxyl-6-methyl-2-pyrone standard as shown in Figure 5, the product was likely to be one of pyrone derivatives. Based on the results, both mutants were also able to utilize hexanoyl-CoA as a starter substrate, however, in this case, both mutants showed a lower production of pyrone product (Figure 6). Generally, the results showed that a single amino acid substitution, particularly the catalytic cysteine 170, affected the enzymatic pyrone production of *Pp*CHS. These changes affecting the production of reaction products might be associated with the alteration in the binding capacity/affinity of the *Pp*CHS catalytic site to the substrates. These results also agreed well with previous work done on chalcone synthase, which suggested that a decrease in or loss of the catalytic activity of the mutants might be caused by the effects of these substitutions on the covalent binding affinity of the starter molecule (hexanoyl-CoA) to the chalcone synthase active site cavity [16].

**Figure 7:**
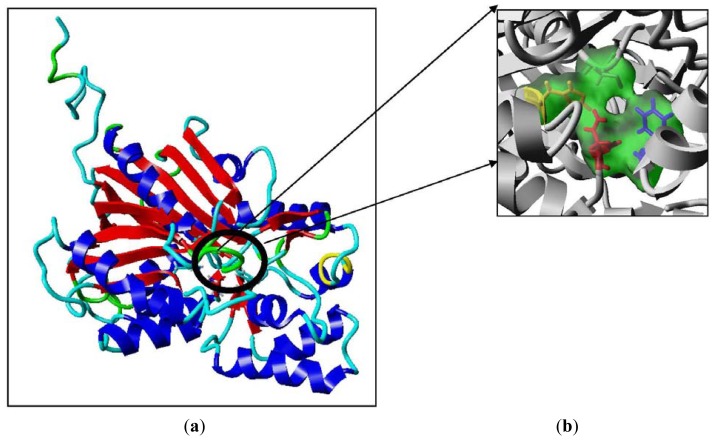
The predicted structure of *Pp*CHS. (**a**) 42 kDa monomer subunit of PpCHS. The predicted structure for *Pp*CHS shown with secondary structure rendered as ribbon; (**b**) *Pp*CHS active site cavity. *Pp*CHS contains catalytic tetrad comprising Cys, 170 (residue shown in green), Phe 221 (residue shown in blue), His 309(residue shown in yellow) and Asn 342 (residue shown in red).

To further examine the validity of this data, homology modeling of *Pp*CHS was performed to provide an insight into the effect of these mutations on the cavity volume of the catalytic site as well as the ion-pair interaction between the substituted residues with the His residue. The three-dimensional structure of *Pp*CHS was built using the crystal structure of chalcone synthase from *Medicago sativa*,, which shares 63% sequence identity. Studies on the three-dimensional model structure of *Pp*CHS revealed that the catalytic residues (Cys, His, Asn, Phe) of polyketide synthase are structurally conserved as illustrated in Figure 7. However, the active site cavity varies which leads to the formation of diverse products and by-products depending on the types of starter molecules used [7]. The calculated distance between the Cys 170 and His 309 as mentioned before is approximately 4 Å. The Cys-His interaction was found to be one of the most crucial points in the reaction mechanism of chalcone synthase.

It has been reported that the cavity volume plays an important role in the strength of the substrate binding interaction and hence any change in it might alter the product formation profile of the type III PKS. Cavity volume plays an important role in the strength of the substrate binding interaction. These cavities are important for molecular recognition to provide a direct connection to the exterior of the protein. The cavity volume of different type III PKS proteins, which includes chalcone synthase, stilbenecarboxylate synthase 2, stilbene synthase and 2-pyrone synthase, were calculated using CASTp, which is an algorithm based on computational geometry methods. It was found that the cavity volume of different type III PKS varied in a range of 737 Å–1683 Å [17].

In this study, the cavity volumes of the mutants (Cys 170 Arg, Cys 170 Ser) were measured using YASARA as listed in Table 1. Interestingly, despite low production of the reaction product by the mutants, the cavity volume for both mutants showed contradicting results in which the Cys 170 Arg mutant’s cavity volume decreased to 195.53 Å while the Cys 170 Ser mutant’s cavity volume increased to 231.23 Å. The substituted arginine residue, due to the presence of a guanidine group, tends to reside in the catalytic cavity of the active site, filling the space between the neighboring residues and leading to a smaller cavity volume of the mutant. However, due to the bulky structure of the guanidine group now occupying the space inside the active site, the positions of neighboring residues residing within the active site as well as the overall structure of the mutant are as shifted, leading to an increase in the Cys 170 Arg mutant’s structure volume to 43,391.02 Å. The increase of the volume of the structure reflects a more flexible and less compact structure which agrees well with the calculated total energy of Cys 170 Arg of −65.03 indicating a decrease of the mutant’s structure stability.

On the other hand, for the substituted serine residue, due to the presence of a smaller hydroxyl group, contributes to a larger space within the catalytic cavity of the active site which is 231.23 Å. Based on the YASARA structure prediction analysis, a hydrogen bond interaction was detected between the hydroxyl group of the Ser 170 and His 309. This interaction along with other possible interactions among other neighboring residues within the active site cavity might be one of the factors contributing to the decrease of the Ser 170 mutant structure volume in comparison with the Arg 170 mutant which is 43,330.84 Å. Therefore, the decrease in volume of the Ser 170 mutant reflects a structure compactness and stability of the whole structure which agrees well with the calculated total energy of −66.97.

This approach was also carried out on stilbene synthase, which evolved from CHS. The mutation of Cys to Ser showed a lower yield of product and an instability of the C164S mutant. Aside from this, other mutational studies carried out to mutate Cys at position 169 with Ser showed failure in detecting enzyme activity which defined the destruction of Cys as an essential role in the catalytic machinery of CHS [18]. However, in this work, some enzyme activity is still detectable by the mutant.

According to the thermal stability profile of recombinant chalcone synthase shown in Figure 4, a decrease in enzymatic activity was observed after pre-incubation (30 min) of enzyme at 35 °C prior to enzyme assay. Surprisingly, both Cys 170 Arg and Cys 170 Ser mutants showed increased enzymatic activity after pre-incubation of the mutant enzymes at 40 °C. Several case studies were done to investigate the effects of mutations on non-active site arginine residues of chalcone synthase. An arginine residue, largely protonated at physiological pH, plays an important role in forming salt bridges with other anionic amino acids. It was found that mutations on Arg 68, Arg 172, and Arg 328 greatly affected the enzyme activity due to their important roles in positioning the active site residues in correct catalytic topology. On the other hand, Arg 199 and Arg 350 were found to play an important role in maintaining the structural integrity and foldability of the enzyme [19].

**Figure 8:**
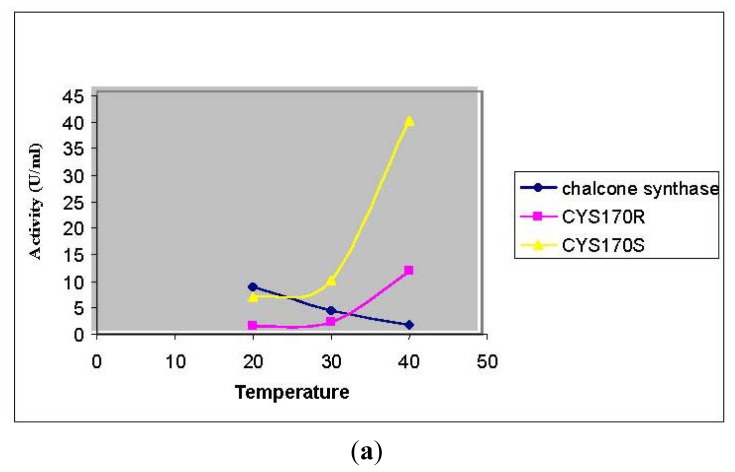

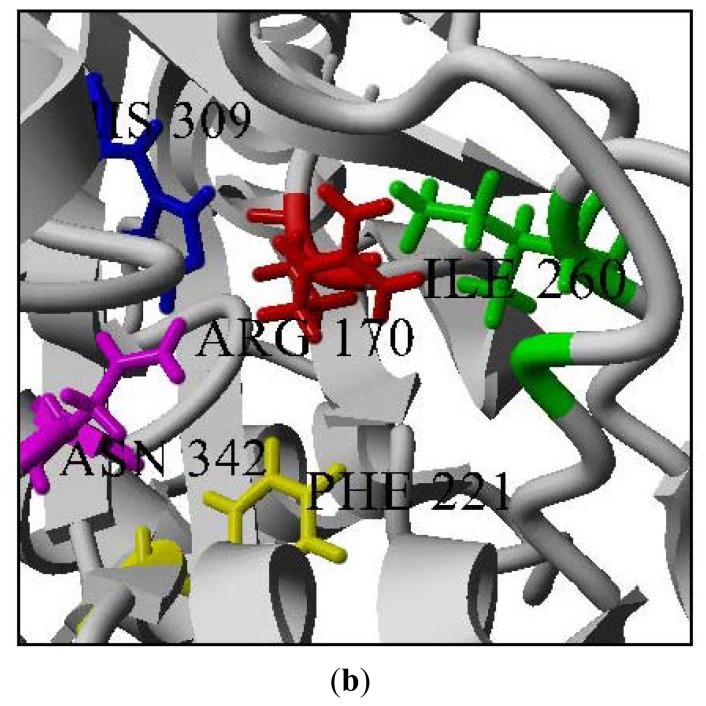
Thermal stability of mutants. (**a**) Thermal stability of *Pp*CHS mutants was carried out in the range of 20 to 40 °C; (**b**) *Pp*CHS active site cavity, *Pp*CHS contains catalytic tetrad comprising Arg170 (residue shown in red), Phe 221 (residue shown in yellow), His 309(residue shown in blue), Asn 342 (residue shown in magenta) and Ile 260 (residue shown in green).

In this study, the Cys 170 Arg mutant exhibited an increase in the thermal stability as is shown in Figure 8(a). Through the homology modeled structure of Cys 170 Arg mutant, there is a possible hydrogen-bonding interaction (1.744 Å) between the Arg 170 residue with Ile 260 located in the middle of a loop, maintaining the rigidity and stability of the whole protein structure (Figure 8(b)). Therefore, improvement in protein stability leads to an increase in the thermal stability of the mutants.

## 3. Materials and Method

### 3.1. Expression and Purification of Recombinant Chalcone Synthase

Cells harboring the recombinant plasmid were cultured in liquid LB medium (200 mL) containing 10 ug/mL ampicillin. When the optical density of the culture at 600 nm reached 0.5, isopropyl-β-d-thiogalactoside (1 mM) was added to the culture to induce recombinant protein expression. After incubation at 37 °C for 12 h, the cells were harvested by centrifugation, resuspended in 10 mL of buffer (20 mM phosphate, pH 7.4 containing 80 mM imidazole and 0.5 M NaCl), and lysed by sonication.

The homogenate was centrifuged at 12,000 rpm for 30 min to remove the insoluble material. Then, the supernatant was applied to a of Ni^2+^ Sepharose Fast Flow column equilibrated with binding buffer (20 mM sodium phosphate buffer, pH 7.4, 500 mM NaCl, 80 mM imidazole). Following a washing step with binding buffer (10 CV), the enzyme was eluted with 300 mM imidazole (20 CV).

### 3.2. Standard Assay Conditions of CHS

Enzyme assays were performed according to [14] with modifications. The standard reaction mixture (500 μL) contained 25 μL of 0.8 mM malonyl-CoA, 25 μL of 0.4 mM hexanoyl-CoA, and 100 μL of enzyme in 20 mM phosphate buffer, pH 7 and the reactions were incubated at 30 °C for 2 h. After termination of the reaction with 1 N HCl, the reaction products were extracted for 2 min with ethyl acetate, and the upper layer was subjected to analysis using HPLC. The reaction products were identified with HPLC (Agilent) using a C18 Hypersil Gold column (5 μm, 4.6 mm × 150 mm) with a flow rate of 0.5 mL/min and using a 280 nm UV detector. The chromatographic separation was performed using a gradient of acetonitrile (30–60% in 30 min) in water.

### 3.3. Characterization of Purified Chalcone Synthase

#### 3.3.1. The Effect of Substrate Specificity

Hexanoyl-CoA or butyryl-CoA was used as the starter CoA in the assay system to determine whether other preferred aliphatic CoAs would yield higher amounts of reaction products. To assess the effects of the substrate concentration on the production of product, hexanoyl-CoA at concentrations of 0.1 mM to 2.4 mM was tested. The chalcone synthase reaction with hexanoyl-CoA was also analyzed every hour for 12 h to determine the time dependence.

#### 3.3.2. The Effect of pH on Chalcone Synthase Activity

The effect of pH on the activity of the recombinant chalcone synthase was measured at various pHs (pH 3–11). The buffer system used was as follows: Fifty milli-moles of acetate buffer (pH 4–6), potassium phosphate buffer (pH 6–8), Tris-Cl buffer (pH 8–9), glycine-NaOH (pH 9–11), and Na2HPO3/NaOH buffer (pH 11–12).

#### 3.3.3. Thermal Stability of Chalcone Synthase Activity

The effect of temperature on the recombinant chalcone synthase was determined at different temperatures ranging from 10 to 60 °C at 10 °C intervals for 2 h. An enzyme stability test was conducted by pre-incubating chalcone synthase at various temperatures ranging from 10 to 60 °C at 5 °C intervals for 30 min prior to performing the chalcone synthase assay at 30 °C for 2 h.

### 3.4. Preparation of CHS Mutants

The C170R and C170S mutants were generated using the QuickChange site-directed mutagenesis kit (Stratagene, USA). Specific mutagenic primers were design to amplify the open reading frame of PpCHS gene.

C170S mutant: (Forward primer 5′-ATG ATG TAC CAA ACC GGG TCT TTC GGC GGT GCA TCC GTG-3′) and (Reverse primer 5′-CAC GGA TGC ACC GCC GAA AGA CCC GGT TTG GTA CAT CAT-3′).

C170R mutant: (Forward primer 5′-ATG ATG TAC CAA ACC GGG AGG TTC GGC GGT GCA TCC GTG-3′) and (Reverse primer 5′-CAC GGA TGC ACC GCC GAA CCT CCC GGT TTG GTA CAT CAT-3′).

After confirmation of the sequences, the plasmids that expressed the mutants were transformed in *E. coli* BL21 (DE3) pLysS respectively.

### 3.5. Protein Expression in *E.coli* and Enzyme Extracts

Both recombinant *Pp*CHS and C170S and C170R mutants were grown to A600~0.5, and induced with 1 mm IPTG for 12 h incubation time. The cells were harvested at 8,000 rpm for 10 min at 4 °C. The pellet was suspended with phosphate buffer prior to sonication and the supernatant collected after centrifugation at 12,000 rpm was used for enzyme activity determination.

### 3.6. HPLC Analysis of Mutant’s Reaction Products

For HPLC analysis, the reaction products were identified using a Nucleosil C18 column with a flow rate of 0.1 mL/min at UV 280 nm detection. The chromatography was run using a gradient of Acetonitrile (30–70%) in water. Under these conditions, 4-hydroxyl-6-methyl-2-pyrone and naringenin were used as an internal standard. The authentic standard of 1 mg/mL of 4-hydroxy-6-metyl-2-pyrone was eluted at a retention time of 1.65 min which is comparable with the formation of mutant’s reaction products. For liquid chromatography-tandem mass spectrometry (LC/MS/MS) detection of unknown compounds, the positive ionization mode was chosen as the detection mode with the collision gas off and was set at 370 °C. The positive ion MRM chromatograms of chalcone synthase reaction product at m/z 272.50–273.50 were obtained from chalcone synthase reaction product containing 100 ng/mL naringenin which further confirms the formation of a chalcone analoque from the reaction products [10].

### 3.7. Sequence Analysis and Homology Modeling

From the output of PSI-BLAST and BLAST in search of a suitable template with a PDB structure, chalcone synthase from *Medicago sativa* with PDB ID: IBQ6 was selected with 63% sequence identity. It is important to obtain the best sequence alignment between the template protein and the model sequence. Therefore, multiple sequence alignments of target sequences with the selected template sequences were performed using structural alignments to establish a baseline for the accuracy of the sequence alignments. Finally, the homology model of *Physcomitrella patens* chalcone synthase (Accession No: DQ 275627.2) was constructed using the Accelerys Discovery Studio suite program.

MODELER Accelrys, San Diego [20] allows the autonomic generation of one or more hypothetical models based upon one or more templates [21]. Once a target-template alignment was constructed, MODELER automatically calculated a 3D model of the target completely. This method generated a *Pp*CHS model structure based on the IBQ6 (*Medicago sativa* chalcone synthase).

#### 3.7.1. Validation and Analysis of Model

The validation and evaluation of the model is an essential process to ensure the accuracy of the generated model. There are many model evaluation programs and servers like the Procheck [22] and the ERRAT [23]. The Procheck is a program that evaluates the initial model and shows in a Ramachandran plot where the percentage amount of residues belong to the core, *i.e.*, in the allowed, generally allowed and disallowed regions of the plot [22]. On the other hand, the ERRAT is a program that allows a quick overview of the structure quality based on a bar graph-like format [24].

The Ramachandran plot for the *Pp*CHS structure represents about 83% of the residues in these cores in the most favored regions. Only 0.6% of the residues are present in the disallowed region. Meanwhile, the evaluation of the *Pp*CHS model structure using ERRAT showed approximately 85% of the sequence below the 95% confidence level limit. For a fully refined structure, 95% of the sequence should fall below the 95% confidence level limit, and the structure was significantly correct. Meanwhile, the sequence showed that the above 99% confidence level was poor [25]. To conclude, the quality of the *Pp*CHS model structure evaluated using PROCHECK and ERRAT is considered to be a reliable predicted structure.

#### 3.7.2. Building Mutants

All the mutants were generated by swapping the point amino acid using the YASARA interface [26]. The predicted native structure and its mutants were selected for further analysis.

In this study, we used the FOLDX algorithm to predict the thermodynamic stability of the mutations. FOLDX is available via web at http://foldx.crg.es [27]. Before running the Foldx executable, a ‘run’.txt file with the commands and options to be used was created and the pdb name was stored in a list.txt file. The FoldX energy function has been found to be important for protein stability. Total energy is the predicted overall stability of the enzyme, while the Van der Waals clashes is the energy penalization due to Van der Waals clashes (interresidue), which are the clashes between atoms from different residues.

Before running the command for stability, the pdb structure needed to first be repaired to optimize the structure and to move all the side chains slightly to eliminate the small Van der Waals’ clashes. The stability calculation was also run with different combinations of mutants. As is shown in Table 2, there are 19 possible positive mutations based on their stability (according to the total energy). However, from the 19 chosen point mutations, Cys 170 Arg (exhibited a lower stability: −65.03) and Cys 170 Ser (exhibited the highest stability: −66.97) were chosen to investigate the different effects of these substitutions on the production of pyrones analogues.

## 4. Conclusions

The recombinant *Physcomitrella patens* chalcone synthase was purified to homogeneity through a single step of affinity chromatography using Ni^2+^ Sepharose and characterized. The work presented here has highlighted the profile of chalcone synthase enzymatic properties based on the production of a product using hexanoyl-CoA as a substrate. The chalcone synthase reaction with hexanoyl-CoA was found to be optimal using Tris-HCl, at a pH 7, at a temperature of 30 °C. The total loss of enzyme activity was observed at 45 °C. It is assumed that the types of products formed by the chalcone synthase reaction with hexanoyl-CoA might be the result of two premature condensation reactions between hexanoyl-CoA and malonyl-CoA. It could be concluded that both Arg 170 and Ser 170 substitutions exhibit important interactions among the other neighboring residues within the catalytic cavity of the active site maintaining the structural integrity and foldability of the enzyme. However, decreases in the enzyme activity by mutants might be related to the fact that these substitutions affect the positioning of the catalytic active site topology in a less favorable manner making it unable to promote subsequent condensation reactions within the catalytic cavity. Therefore, these results suggest that the overall protein dynamics of chalcone synthase is affected even by a single point mutation.

## Figures and Tables

**Table 1:** Comparisons of the *Pp*CHS mutant’s dyad interactions and cavity volume.

Type/Mutants	Dyad Interactions (Distance)	Cavity Volume
**Recombinant** ***Pp*CHS**	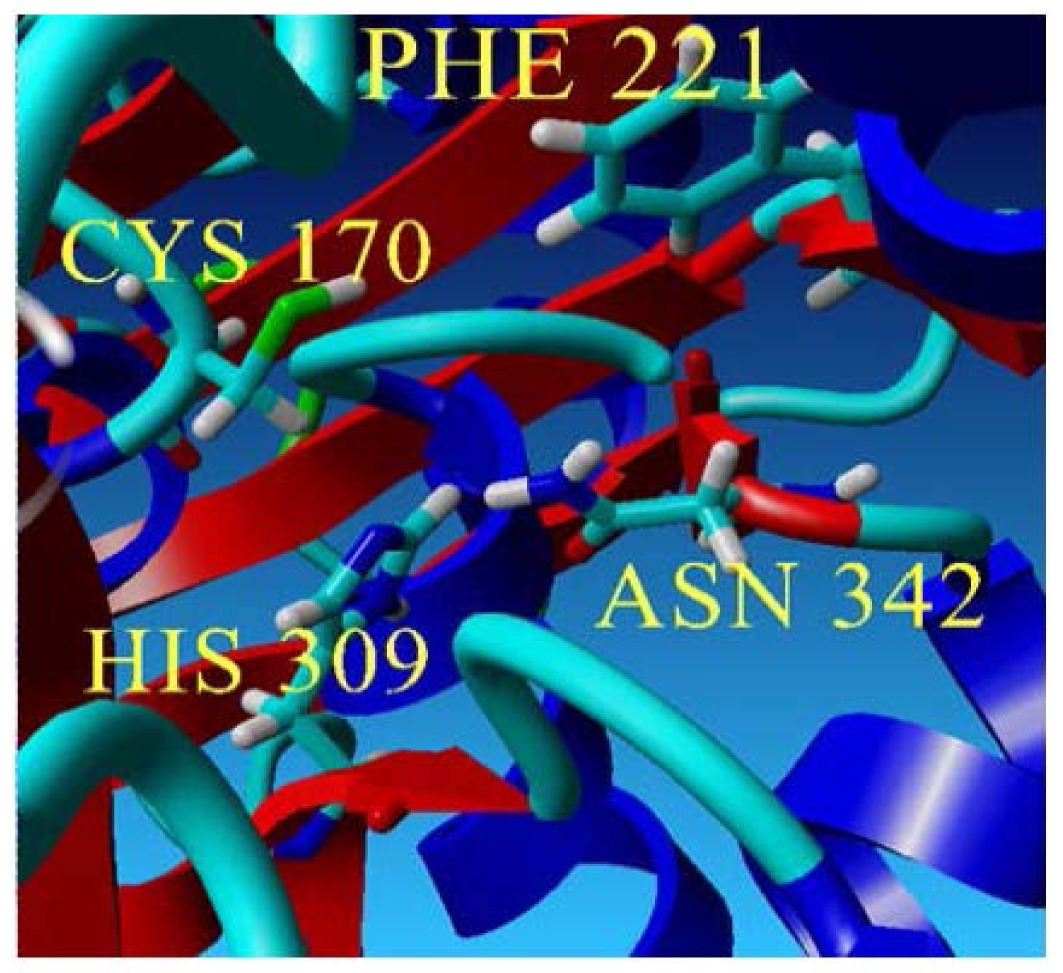 Cys-His : 4.0 Å	216.31 Å^3^
**Cys 170 Arg Mutant**	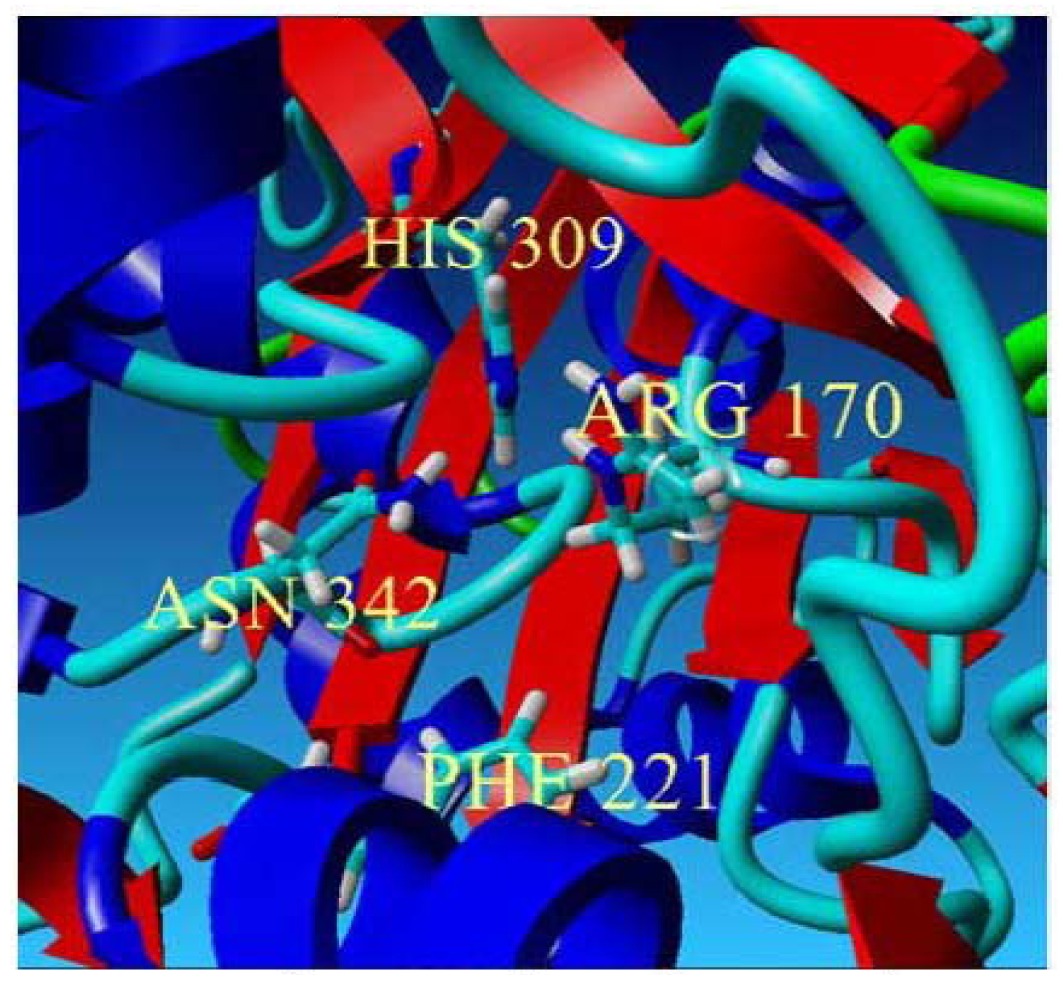 No interaction detected	195.53 Å^3^
**Cys 170 Ser Mutant**	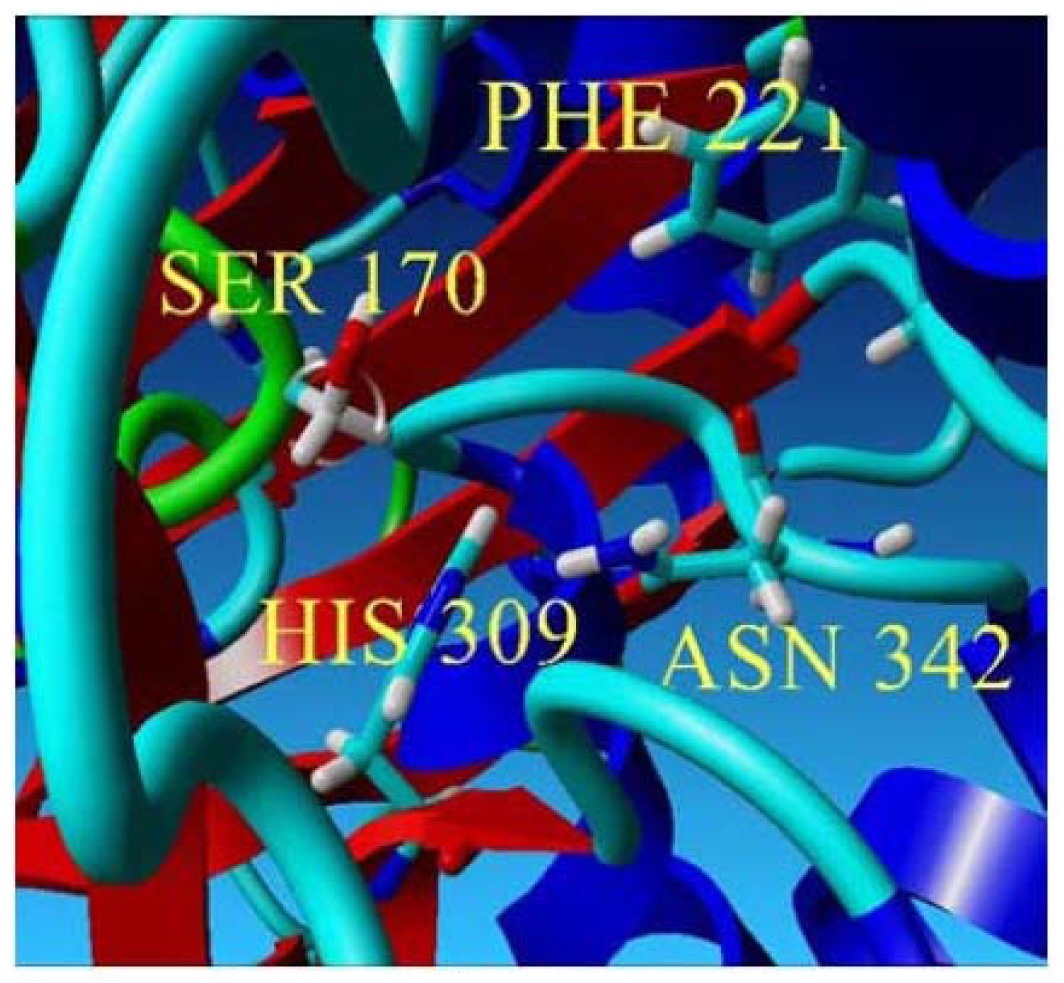 Ser-His : 3.931 Å	231.23 Å^3^

**Table 2:** Total energy (kcal/mol) with other free energy of wild-type and its mutants by FOLDX of *Pp*CHS.

Substitutions (Residues)	Total Energy (kcal/mol)
Wild type reference (Cys 170)	−66.62
Cys 170 Gly mutant	−66.72
Cys 170 Ala mutant	−66.87
Cys 170 Leu mutant	−66.78
Cys 170 Val mutant	−65.54
Cys 170 Ile mutant	−65.83
Cys 170 Pro mutant	−69.28
Cys 170 Arg mutant	−65.03
Cys 170 Thr mutant	−65.59
Cys 170 Ser mutant	−66.97
Cys 170 Met mutant	−68.13
Cys 170 Lys mutant	−65.53
Cys 170 Glu mutant	−67.44
Cys 170 Gln mutant	−67.05
Cys 170 Asp mutant	−66.72
Cys 170 Asn mutant	−65.68
Cys 170 Trp mutant	−63.97
Cys 170 Tyr mutant	−66.45
Cys 170 Phe mutant	−66.07
Cys 170 His mutant	−64.15
